# Colour M-mode superiority in evaluation of improvement in myocardial performance indices following successful percutaneous coronary intervention (PCI)

**DOI:** 10.5830/CVJA-2010-061

**Published:** 2011-08

**Authors:** R Sattarzadeh, M Maleki, A Jamalian, A Amirpour, A Firuzi, N Samiei, M Esmaeilzadeh, A Ghorbani, A Tavoosi

**Affiliations:** Department of Cardiology, Tehran University of Medical Sciences, Imam Khomeini Hospital, Tehran, Iran; Department of Cardiology, Shaheed Rajaie Cardiovascular Medical and Research Centre, Iran Medical University, Tehran, Iran; Department of Cardiology, Shaheed Rajaie Cardiovascular Medical and Research Centre, Iran Medical University, Tehran, Iran; Department of Cardiology, Shaheed Rajaie Cardiovascular Medical and Research Centre, Iran Medical University, Tehran, Iran; Department of Cardiology, Shaheed Rajaie Cardiovascular Medical and Research Centre, Iran Medical University, Tehran, Iran; Department of Cardiology, Shaheed Rajaie Cardiovascular Medical and Research Centre, Iran Medical University, Tehran, Iran; Department of Cardiology, Shaheed Rajaie Cardiovascular Medical and Research Centre, Iran Medical University, Tehran, Iran; Digestive Diseases Research Centre, Tehran University of Medical Sciences, Shariati Hospital, Tehran, Iran; Department of Cardiology, Tehran University of Medical Sciences, Imam Khomeini Hospital, Tehran, Iran

**Keywords:** percutaneous coronary intervention, echocardiography, coronary artery disease, systolic function, diastolic function

## Abstract

**Aim:**

This study aimed at evaluating the early effects of successful elective percutaneous coronary intervention (PCI) on systolic and diastolic function.

**Methods:**

We consecutively studied the systolic and diastolic function in 21 patients with stable coronary artery disease (CAD) and left ventricular ejection fraction (LVEF) > 40% before and 48 hours after successful elective PCI.

**Results:**

Tei index and systolic indices (LVEF, regional wall motion abnormality score, tricuspid annular plane systolic excursion and peak systolic velocity of mitral and tricuspid annulus) did not change significantly. Among the diastolic indices, only velocity propagation (Vp) improved significantly (from 42.9 ± 10.8 to 51.8 ± 10.7, *p*-value = 0.008) following PCI. Diastolic velocities, E/A ratio, deceleration time (DT), early and late diastolic velocities of mitral annulus in TDI, pulmonary vein systolic (PVs) and diastolic flow velocity (PVd) did not show significant improvement.

**Conclusion:**

Propagation velocity of mitral inflow was the earliest index to recover following successful PCI in patients with stable CAD.

## Abstract

Percutaneous coronary intervention (PCI) is a very common revascularisation procedure performed in patients with stable coronary artery disease (CAD).^1^ PCI has shown promise in terms of reduction of symptoms and improvement in quality of life.^2^,^3^ However, physiological cardiac changes following improvement of anatomical blood flow after PCI are still unclear.

Echocardiography provides a feasible and non-invasive technique for assessment of global and regional myocardial function.^4^ Results of echocardiographic assessment of systolic and diastolic ventricular function following successful elective PCI have been contradictory and confusing.^5^-^9^ However, the techniques used in previous studies were mainly based on qualitative and semi-quantitative assessment of myocardial function, and information on quantification of regional and global myocardial function with the recently introduced technique, tissue Doppler imaging (TDI),^10^-^12^ following PCI is limited.

The present study was designed to evaluate the early effects of successful elective PCI on systolic and diastolic function globally and regionally through thorough echocardiographic examinations, including two-dimensional (2-D), M-mode, colour Doppler and TDI.

## Methods

Our study participants were recruited from 31 consecutive patients with stable CAD who were scheduled for elective PCI in Rajaie Heart Centre. Patients were considered for inclusion if they had angiographically documented coronary artery stenosis > 70% in diameter in the culprit lesion by visual assessment, and documented ischaemia. Ischaemia was documented using stress testing in patients with typical stable angina, according to the Canadian Cardiovascular Society (class I or II).

Exclusion criteria were bundle branch block, pacemaker implantation, left ventricular ejection fraction (LVEF) < 40%, valvular heart disease, cardiomyopathy, history of coronary artery bypass grafting (CABG), refractory angina or acute myocardial infarction (MI) requiring emergency revascularisation, and left main stem disease.

All patients were evaluated by an experienced echocardiologist, using conventional (2-D, M-mode and colour Doppler) and tissue Doppler echocardiography examinations one day before and 48 hours after successful PCI, in order to assess cardiac systolic and diastolic function. Successful revascularisation was defined as a residual stenosis of < 30% in luminal diameter with TIMI flow grade 3. Patients with unsuccessful PCI (10 people) were excluded.

All patients were on an optimal medical regimen consisting of nitrates, aspirin, β-blockers, angiotensin converting enzyme inhibitors and lipid-lowering agents (statins) along with a low-fat diet on an individual basis.

The institutional review board of the hospital approved the study protocol. All participants gave written informed consent. This investigation was in accordance with the Declaration of Helsinki.

Direct stenting or stenting after successful angioplasty was performed in all the participants according to published guidelines.^13^ All patients received heparin to a target activated thrombin time level of 200–300 sec, and also clopidogrel in standard doses.

Echocardiographic data were obtained using a commercially available ultrasound system Vivid-3 (GE) equipped with a 3-MHz transducer. Measurements were obtained by averaging values recorded in three consecutive cycles, according to the current American Society of Echocardiography guidelines.^14^

For two-dimensional and M-mode echocardiography, 2-D images were obtained in standard parasternal and apical views. All patients were examined at rest in the left lateral decubitus position.

The LV end-diastolic and end-systolic dimensions (LVEDD and LVESD) were measured at the level of the mitral leaflet tips from the parasternal long-axis with two-dimensional targeted M-mode echocardiography. LVEF was calculated from the apical two- and four-chamber views using the modified Simpson’s rule.

The regional wall motion abnormality (RWMA) score of the LV was assessed for each LV segment individually, and the wall motion abnormality score index (WMSI) was obtained using the 16-segment model, according to the American Society of Echocardiography recommendations.^14^ Higher scores were considered for more severe wall motion abnormalities (1: normal, 2: hypokinesis, 3: akinesis, 4: dyskinesis, 5: aneurysmal).

For RV systolic functional assessment, the tricuspid annular plane systolic excursion (TAPSE) was measured using two-dimensionally guided M-mode imaging from the apical four-chamber view.

Mitral inflow velocities were measured with pulsed-wave colour Doppler echocardiography, with the sample volume positioned between the tips of the mitral leaflets in the apical four-chamber view. Peak early diastolic velocity (E), peak late diastolic velocity (A), E/A ratio and deceleration time (DT) were obtained.

Mitral inflow propagation velocity (Vp) was measured as the maximum slope of the first aliasing velocity during early filling from the mitral valve plane to 4 cm distal to the LV cavity in the apical four-chamber view using colour M-mode Doppler.

Pulmonary vein systolic flow velocity (PVs) and diastolic flow velocity (PVd) were measured from the apical four-chamber by placing a sample volume in the right upper pulmonary vein with Doppler echocardiography. Isovolumetric contraction time (IVCT), isovolumetric relaxation time (IVRT) and ejection time (ET) were assessed by simultaneously measuring the flow into the LV outflow tract and the mitral inflow, using Doppler echocardiography. The index of myocardial performance (IMP or Tei index) was calculated by dividing the sum of IVRT and IVCT by ET.

Pulsed-wave TDI was performed by activating the tissue Doppler function in the same echocardiographic machine. Mitral annulus velocities (myocardial systolic and diastolic velocities) were measured using the pulsed-wave TDI technique, by placing a 1–2-mm sample volume at the levels of septal, lateral, inferior, anterior and posterior annulus.

Peak systolic velocity of the mitral annulus (Sa), and early (Ea) and late diastolic (Aa) velocities of the mitral annulus were determined from septal, lateral, inferior, anterior and posterior aspects. Peak systolic velocity of the tricuspid annulus (TA-Sa) was also derived from pulsed Doppler tissue imaging of the lateral tricuspid annulus.

## Statistical analysis

Continuous variables are presented as means ± standard deviations (SD) and categorical data are expressed as frequencies and percentages. Comparisons of continuous variables pre and post PCI were carried out using the paired-samples *t*-test. The Wilcoxon signed rank test was used to test for the difference between the mean RWMA scores before and after PCI. The statistical software SPSS-13 (Chicago, IL, USA) for Windows was used for all analyses; *p*-values < 0.05 were considered statistically significant.

## Results

A total of 21 patients who had had successful PCI were considered for the study analysis. Baseline characteristics of participants are summarised in Table 1. LVEF, RWMI and peak systolic velocities remained unchanged 48 hours following elective PCI (Table 2). The index of myocardial performance did not change (Table 2). Vp improved significantly early after PCI (*p* = 0.008). Other diastolic indices did not show improvement (Table 3).

**Table 1.** Baseline Characteristics Of The Patients

	Study population (n = 21)*
Age (years)	54.05 ± 10.08
Male (%)	14 (66.6)
BMI (kg/m^2^)	25.72 ± 3.97
Heart rate	76.05 ± 11.36
LVESD (cm)	3.27 ± 0.53
LVEDD (cm)	5.03 ± 0.42
Angiographic results (%)	
Mono-vessel involvement	12 (57.1)
Two-vessel involvement	6 (28.6)
Three-vessel involvement	3 (14.3)

*Means ± standard deviation. BMI: body mass index; LVESD: left ventricular end-systolic diameter; LVEDD: left ventricular enddiastolic diameter.

**Table 2.** Early Echocardiographic Indices Of Ventricular Systolic Function Before And After Successful PCI In 21 Patients With Stable CAD*

*Method*	*Index*	*Pre-PCI*	*Post-PCI*	p*-value*
2-D echocardiography				
	LVEF (%)	47 ± 7.7	48.4 ± 9.2	0.10
	WMSI	1.41 ± 0.35	1.39 ± 0.39	0.26
M-mode echocardiography				
	TAPSE (mm)	23 ± 2.7	23.2 ± 3.2	0.64
Doppler echocardiography				
	Tei index	0.4 ± 1.1	0.4 ± 1.1	0.33
Tissue Doppler Imaging (cm/sec)				
	Sa – septal	7.10 ± 1.37	7.29 ± 1.42	0.58
	Sa – lateral	8.52 ± 2.25	8.19 ± 2.36	0.44
	Sa – inferior	8.38 ± 1.91	8 ± 1.30	0.29
	Sa – anterior	7.48 ± 2.14	7.2 ± 1.47	0.46
	Sa – posterior	8.19 ± 1.72	8.24 ± 1.58	0.92
	TA-Sa	11.8 ± 2	12.2 ± 2	0.282

*Means ± standard deviation. PCI: percutaneous coronary intervention; CAD: coronary artery disease; LVEF: left ventricular ejection fraction; WMSI: wall motion abnormality score index; Sa: peak systolic velocity of mitral annulus; TA-Sa: peak systolic velocity of tricuspid annulus.

**Table 3.** Early Echocardiographic Indices Of Ventricular Diastolic Function Before And After Successful PCI In 21 Patients With Stable CAD*

*Method*	*Index*		*Pre-PCI*	*Post-PCI*	p*-value*
Doppler echocardiography					
	E (m/s)		0.7 ± 0.2	0.7 ± 0.2	0.52
	A (m/s)		0.7 ± 0.2	0.7 ± 0.2	0.83
	E/A		1 ± 0.4	0.9 ± 0.2	0.27
	DT (m sec)		214.1 ± 50	204.7 ± 57.8	0.48
	IVRT (m sec)		93.6 ± 18.7	96.3 ± 18.5	0.53
	Vp (cm/sec)		42.9 ± 10.8	51.8 ± 10.7	0.008
	PVs (cm/sec)		55.2 ± 11	56.9 ± 11.7	0.41
	PVd (cm/sec)		44 ± 10.4	42 ± 2.3	0.46
Tissue Doppler imaging (cm/sec)					
	Septal	Ea	6.6 ± 1.7	7.3 ± 2.2	0.08
		Aa	8.8 ± 1.7	8.8 ± 1.7	1.00
	Lateral	Ea	9 ± 2.8	8.5 ± 2.6	0.29
		Aa	8.2 ± 2.4	8.4 ± 1.9	0.79
	Inferior	Em	7.6 ± 2.9	7 ± 1.5	0.32
		Aa	7.6 ± 1.5	7.8 ± 2.1	0.72
	Anterior	Ea	8 ± 2.8	7.3 ± 2.6	0.14
		Aa	10 ± 2.4	9.8 ± 2	0.75
	Posterior	Ea	8.5 ± 3.1	8.5 ± 2.8	0.94
		Aa	9.4 ± 3.3	8.2 ± 2.3	0.08

*Means ± standard deviation. TAPSE: tricuspid annular plane systolic excursion; E: peak early diastolic velocity; A: peak late diastolic velocity; DT: deceleration time; IVRT: isovolumic relaxation time; Vp: mitral inflow propagation velocity; PVs: pulmonary vein systolic flow velocity; PVd: pulmonary vein diastolic flow velocity; Ea: early diastolic velocitiy of mitral annulus; Aa: late diastolic velocitiy of mitral annulus.

## Discussion

This study presents a comprehensive echocardiographic assessment of regional and global myocardial function, both systolic and diastolic, following blood flow restoration with PCI electively performed in patients with stable CAD. Our findings indicate that the propagation velocity of early flow into the LV cavity (Vp), measured by colour M-mode Doppler, was the most sensitive index to recover after successful PCI.

LV ejection fraction calculated by the biplanar Simpson’s method, along with other parameters of myocardial systolic function including the regional wall motion index evaluated by 2-D and TDI-derived systolic myocardial velocities at the mitral (Sa) and tricuspid annulus (TA-Sa), did not change significantly early after PCI. Previous studies have shown different results regarding systolic functional improvement following PCI. In a study performed in 41 patients with stable CAD, successful elective PCI did not improve ejection fraction at the one-day and six-month follow up.^5^

However, in another study, peak systolic velocities of three annular sites, but not the ejection fraction, showed substantial improvement.^6^ It was also shown in 32 patients that resting EF and WMSI improved significantly after three to five days and six months, following successful elective PCI.^7^ In addition, the index of myocardial performance (Tei index), which reflects both systolic and diastolic myocardial performance, did not improve in our study.

It is postulated that patients with severely depressed ventricular dysfunction (LVEF < 40%) benefit most from revascularisation.^15^ Therefore the possible explanation for these findings could be the fact that the patients included in our study had LVEF > 40% at baseline and therefore experienced little improvement.

Among various echo-technique parameters assessing diastolic function, only colour M-mode Vp was found to have improved significantly 48 hours after PCI. There have been various reports on improvements observed in diastolic parameters following PCI. Diller *et al*.^16^ analysed the results of successful elective PCI, using TDI before PCI as well as one day and six weeks after the procedure, on systolic and diastolic function in 24 consecutive patients with CAD and normal systolic function. They found that all tissue Doppler measurements of early diastolic function improved significantly after PCI, and systolic peak velocity improved in the septal, lateral, inferior and right ventricular areas.^16^

A study performed on 31 patients with chronic CAD showed that most echocardiographic diastolic indices, including IVRT, EDT, E/A ratio, IVCT and diastolic indices of both the mitral and tricuspid annulus sites on TDI improved significantly 24 hours following elective PCI.^6^ In another study, LV diastolic filling improved marginally within 10 days of PCI and persisted up to 30 days.^8^ By contrast, in a study in 15 patients with single anterior descending coronary artery stenosis and normal systolic function, abnormalities in early and late LV filling velocities lasted as long as three months after PCI.^9^

There is evidence that abnormalities in diastolic function may precede systolic dysfunction during myocardial ischaemia.^17^ It has been shown that early LV diastolic function becomes severely impaired during ischaemia.^18^ The early ventricular relaxation is a highly energy-dependent process.^19^ Therefore, the restoration of blood flow following PCI might affect diastolic function earlier than systolic function.

Vp, which was initially proposed by Brun *et al*.^20^ as a sensitive non-invasive indicator of impaired diastolic relaxation, represents the rate of change in velocity of blood flow in early diastole and indicates the time difference between maximal velocity at the apex level and the mitral leaflet tips. It is a unique measure for accurate and reliable assessment of ventricular relaxation, since it is independent of preload.^21^

The reason for persistence of abnormal indices of LV performance (other than Vp) may be the duration of disease before PCI.^22^ These indices may have been improved if patients had undergone PCI earlier during the course of their disease. Hence we say ‘time is muscle’. However, it is believed, based on the ‘open artery theory’, that even late reperfusion provides beneficial effects on cardiac function.^23^ It has been shown, however, that myocardial performance improves gradually after PCI,^24^ so we are hopeful that further improvements in myocardial function will be achieved over time following successful PCI.

A limitation to the current study could have been the relatively small number of patients evaluated. Moreover, since functional improvement following PCI may increase over time,^24^ early assessment of myocardial performance could be another limitation of this study. The final shortcoming may have been the fact that we did not consider clinical improvement along with echocardiographic parameters. To our knowledge, this study is, however, the first to cover a wide range of echocardiographic indices for post-PCI functional assessment in patients with stable CAD.

## Conclusion

Unlike studies that showed an improvement in TDI parameters of systolic and diastolic function in patients with stable CAD after successful PCI, in our population only the colour M-mode Doppler parameter improved 48 hours after successful elective PCI in patients with stable CAD. More studies should be done to corroborate the results presented here.
